# Genome Reshuffling for Advanced Intercross Permutation (GRAIP): Simulation and Permutation for Advanced Intercross Population Analysis

**DOI:** 10.1371/journal.pone.0001977

**Published:** 2008-04-23

**Authors:** Jeremy L. Peirce, Karl W. Broman, Lu Lu, Elissa J. Chesler, Guomin Zhou, David C. Airey, Amanda E. Birmingham, Robert W. Williams

**Affiliations:** 1 Center for Neuroscience, Department of Anatomy and Neurobiology, University of Tennessee Health Science Center, Memphis, Tennessee, United States of America; 2 Center for Genomics and Bioinformatics, Department of Anatomy and Neurobiology, University of Tennessee Health Science Center, Memphis, Tennessee, United States of America; 3 Department of Biostatistics, Johns Hopkins University, Baltimore, Maryland, United States of America; 4 Dharmacon, Inc., Lafayette, Colorado, United States of America; Centre National de la Recherche Scientifique, France

## Abstract

**Background:**

Advanced intercross lines (AIL) are segregating populations created using a multi-generation breeding protocol for fine mapping complex trait loci (QTL) in mice and other organisms. Applying QTL mapping methods for intercross and backcross populations, often followed by naïve permutation of individuals and phenotypes, does not account for the effect of AIL family structure in which final generations have been expanded and leads to inappropriately low significance thresholds. The critical problem with naïve mapping approaches in AIL populations is that the individual is not an exchangeable unit.

**Methodology/Principal Findings:**

The effect of family structure has immediate implications for the optimal AIL creation (many crosses, few animals per cross, and population expansion before the final generation) and we discuss these and the utility of AIL populations for QTL fine mapping. We also describe Genome Reshuffling for Advanced Intercross Permutation, (GRAIP) a method for analyzing AIL data that accounts for family structure. GRAIP permutes a more interchangeable unit in the final generation crosses – the parental genome – and simulating regeneration of a permuted AIL population based on exchanged parental identities. GRAIP determines appropriate genome-wide significance thresholds and locus-specific P-values for AILs and other populations with similar family structures. We contrast GRAIP with naïve permutation using a large densely genotyped mouse AIL population (1333 individuals from 32 crosses). A naïve permutation using coat color as a model phenotype demonstrates high false-positive locus identification and uncertain significance levels, which are corrected using GRAIP. GRAIP also detects an established hippocampus weight locus and a new locus, *Hipp9a*.

**Conclusions and Significance:**

GRAIP determines appropriate genome-wide significance thresholds and locus-specific P-values for AILs and other populations with similar family structures. The effect of family structure has immediate implications for the optimal AIL creation and we discuss these and the utility of AIL populations.

## Introduction

The often-striking variation in heritable traits is usually produced by a multitude of polymorphic genes and a variety of environmental factors. Quantitative trait locus (QTL) mapping provides an effective approach to localizing regions of the genome that are likely to contain modifiers of the phenotype. Coarse mapping of a QTL to a 15–30 cM interval has become a relatively routine matter in traits with at least moderate heritability [1], [2], but fine mapping–narrowing the QTL interval to include only a few candidate genes–is still a much more challenging task. Numerous genetic strategies for narrowing QTL intervals have been attempted with varying degrees of success.

Advanced intercross lines (AILs), first introduced by Darvasi and Soller [3], are one such strategy that is capable of producing a population able to narrow mapping intervals for all QTLs for a given trait at once, given genotype information across the relevant intervals. Since their theoretical introduction, [3] AIL populations have been used by several groups to refine the positions of QTLs in mice [4]–[8]. The statistical analysis in each case that we are aware of has been a standard mapping method designed for application to intercross (F2), backcross (N2), or recombinant inbred (RI) and similar populations where each individual (or strain in the case of RIs) can be treated as an independent observation.

**Figure 1:**
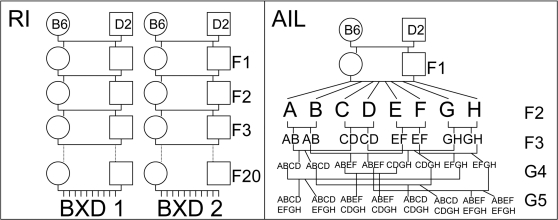
RI and AIL breeding schemes. The left panel of this figure diagrams breeding of a small recombinant inbred (RI) strain set. Each strain is essentially a set of repeated intercrosses starting with inbred parental strains. The animals are considered inbred at 20 generations. The right panel is an example of breeding an advanced intercross line. (Typically such lines consist of 50–100 animals at each generation rather than the 8 shown.) Letters A–H indicate 8 unique F2 animals, and their offspring “inherit” these identifiers. Breeding pairs are chosen for minimum relatedness at each generation.

An AIL (Fig. 1, right panel) is generated by intercrossing a population of F2 animals to generate an F3 population. Members of the F3 population are themselves bred to create the fourth generation of the cross (G4) and so on, either by randomizing the choice of breeding pairs or by selecting the least related pairs for breeding at each generation in order to minimize fixation. Note that beyond F3, mating is not filial, which is indicted by the use of “G” for subsequent generations.

Given the complex breeding history of an AIL populations, however, the assumption of independence is formally incorrect, and this can become a serious problem depending on the details of the construction of the testing population (N) from the breeding population (N_b_), following Darvasi and Soller's nomenclature. Darvasi and Soller consider populations where N = N_b_ as well as populations where a large number of animals (N) are derived from a moderate N_b_. It is with variations on the latter population structure that we are concerned here since populations where N = N_b_ quickly become impractical given space and funding constraints.

Darvasi and Soller's simulation assumes that when expanding N_b_ to generate N animals for testing, one offspring is taken from each of N crosses and tested (A. Darvasi, personal communication). In practice, AIL populations generated in the lab have generally been expanded in the final generation either by generating multiple litters from the same crosses (the UTHSC cross described below for example), or by retaining a larger number of animals in the penultimate generation and using this expanded set of animals to produce one litter per cross [6], [9]. In the AIL population generated at UTHSC, for instance, there are many offspring (41±20; range 4–80 per cross) derived from 32 crosses in the final generation, with wide variation in family size. We will refer to the offspring of each cross generically as a family, but we are particularly concerned with the families resulting in the final generation, in our case the G10 generation resulting from a G9xG9 cross. Because there are few families, each with many members, use of an analysis method that deals appropriately with family relatedness and non-syntenic association in an AIL is crucial. Despite rotation or expansion of parents in the final generation, families will vary dramatically in their relatedness to each other and in the extent of fixation and non-syntenic association within each family and between closely related families based on the relatedness of particular breeding pairs.

While family structure can probably be ignored if only one animal per cross is tested and there are a large number of crosses, there are several serious problems that result from neglecting family structure in AILs when multiple animals per family make up the N tested animals. First, simply shuffling genotypes and phenotypes as can be done with a population of genetically independent individuals constitutes over-randomization in an AIL and gives low genome-wide significance thresholds. Consider an analogous population—RIs. RI animals are fully inbred extended intercrosses (Fig. 1, left panel) usually formed from two or more inbred strains. Phenotyping a large population of several recombinant inbred strains is similar to the AIL situation in that the effective number of independent observations is smaller than the number of animals, because within strain the RIs are highly related (identical, in fact) to each other. This is similar to the observation that AILs in the same family are more highly related to each other than to AIL offspring in other families. This analogy is not complete in that AIL offspring between families are not independent while different RIs, at least those generated by pure repeated intercrosses from inbred strains, are independent.

The second issue is non-syntenic association of markers within the offspring of each cross. In each family, assuming a 1:1 ratio of alleles in the population, which is the best-case scenario, 12.5% of loci will be fixed in both parents and thus in all offspring [10]. If a region containing a gene that affects the phenotype is fixed, all of the other fixed loci in that family will be in disequilibrium with the phenotypic variation, resulting in bias of particular loci and in false positive QTLs, especially where the number of families is small. While drift exacerbates this problem in AILs, it is the same problem that would be present in a population of F3 animals if more than one F3 from a particular F2×F2 cross were phenotyped and analyzed without respect to pedigree. (In such a population, however, a granddaughter design [11] could be used for analysis if the F2 genotype were known.) Of course, chance genotype correlations will exist in independent populations as well, but their strength will be rapidly reduced as the number of independent individuals increases. Likewise, a larger number of families in the final AIL generation will reduce the impact of this problem.

A third problem is that there are likely to be different numbers of animals per family. There might be five litters of a particularly fecund family and one or two litters of a less fecund one if breeders are not rotated regularly, for instance, as was the case in our G9xG9 expansion to generate our G10 generation for testing. The genotype correlations present in larger families will have a greater effect on the mapping outcome than those present in smaller families.

Another way to think about this problem is to take it to its logical extreme – a set of RI lines. Typically in an RI analysis the phenotypes of a number of RI animals of the same strain are averaged and the mean is used as the strain's phenotype, so each paired phenotype and genotype are independent. If the individual observations were used directly, instead, as would be the case with an F2 population—one entry for each animal rather than each strain, randomization by shuffling will obliterate the within-strain structure, resulting in overly low significance thresholds, and even small non-syntenic correlations would be exacerbated dramatically. This would be even more extreme than in an AIL because the strains are fully inbred so fixed loci are more prevalent. If different numbers of animals are selected to represent each strain's offspring, using the individual observations directly would also mean that a more fecund RI strain would have a much larger influence on the QTL map than a less fecund strain despite the fact that each represents only one genome.

When evaluating the utility of AIL populations for fine mapping QTLs when genotyped in a narrow interval and for nominating new QTLs in a genome-wide scan, it is important to have an understanding of the ways in which family structure affects nominal significance at arbitrary points on the genome. Assumptions of independence are violated in AIL populations, and non-syntenic association can cause occurrence of nominally significant results to be more frequent than expected. When using selectively genotyped AIL populations for fine mapping purposes this is particularly problematic since genome-wide changes in the distribution of nominal significance measures will not be readily observable.

**Figure 2:**
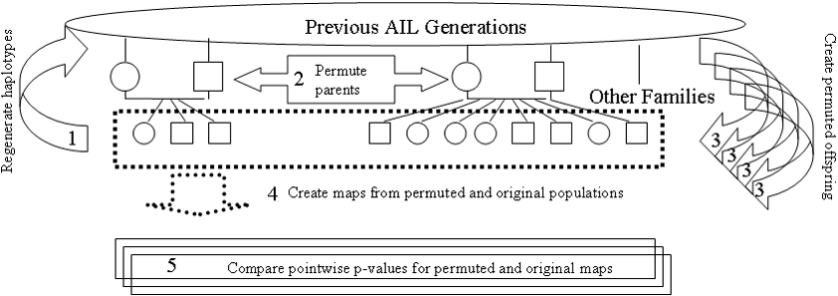
The GRAIP approach. This cartoon summarizes the GRAIP approach. First (1) parental happlotypes are regenerated if they are not already known and (2) the parents are permuted. Next (3) the population of offspring is regenerated using the permuted genotypes and (4) permuted maps are generated using the non-permuted phenotypes. Finally, (5) the significance of the permuted maps are compared at a pointwise and whole genome basis with the original map.

In order to appropriately address these challenges we developed GRAIP, (Fig. 2) an approach that interchanges genomes in the parents of the final cross of an AIL and appropriately simulates the final generation. The GRAIP approach also calculates P-values by locus to compensate for the non-random distribution of alleles at each locus. We describe an application of this method to a large (N = 1333 animals) AIL population developed at the University of Tennessee Health Science Center (UTHSC) and densely genotyped at 329 markers in the expanded population of the final generation. This extensively genotyped resource (most AIL populations are only genotyped near known QTL regions) facilitates generation of genome-wide QTL maps using an AIL population and allows us to evaluate the potential of AILs, when analyzed with approaches that account for family structure, for discovery of new QTLs in addition to fine mapping of known loci. By estimating the proportion of the genome above a given LOD score, we also evaluate the likelihood of mistakenly identifying signal in a QTL region when the strength of association at most markers is unknown, as is the case in most AIL-based studies.

## Results

A direct comparison of GRAIP with a naïve permutation (randomization of phenotypes without respect to family structure) is shown below for two sample populations: an AIL and a set of RI strains taken as individual observations using coat color as a phenotype. Coat color is a well-characterized oligogenic trait for which all loci that segregate between B6 and D2 are known. We also present a comparison of GRAIP and naïve permutation for hippocampus weight, which is a far more polygenic phenotype that has been previously but not exhaustively characterized. While not all determinants for hippocampus weight are known, it is a good model of a more polygenic trait.

### Comparison of naïve permutation and GRAIP using coat color as a model phenotype

**Figure 3:**
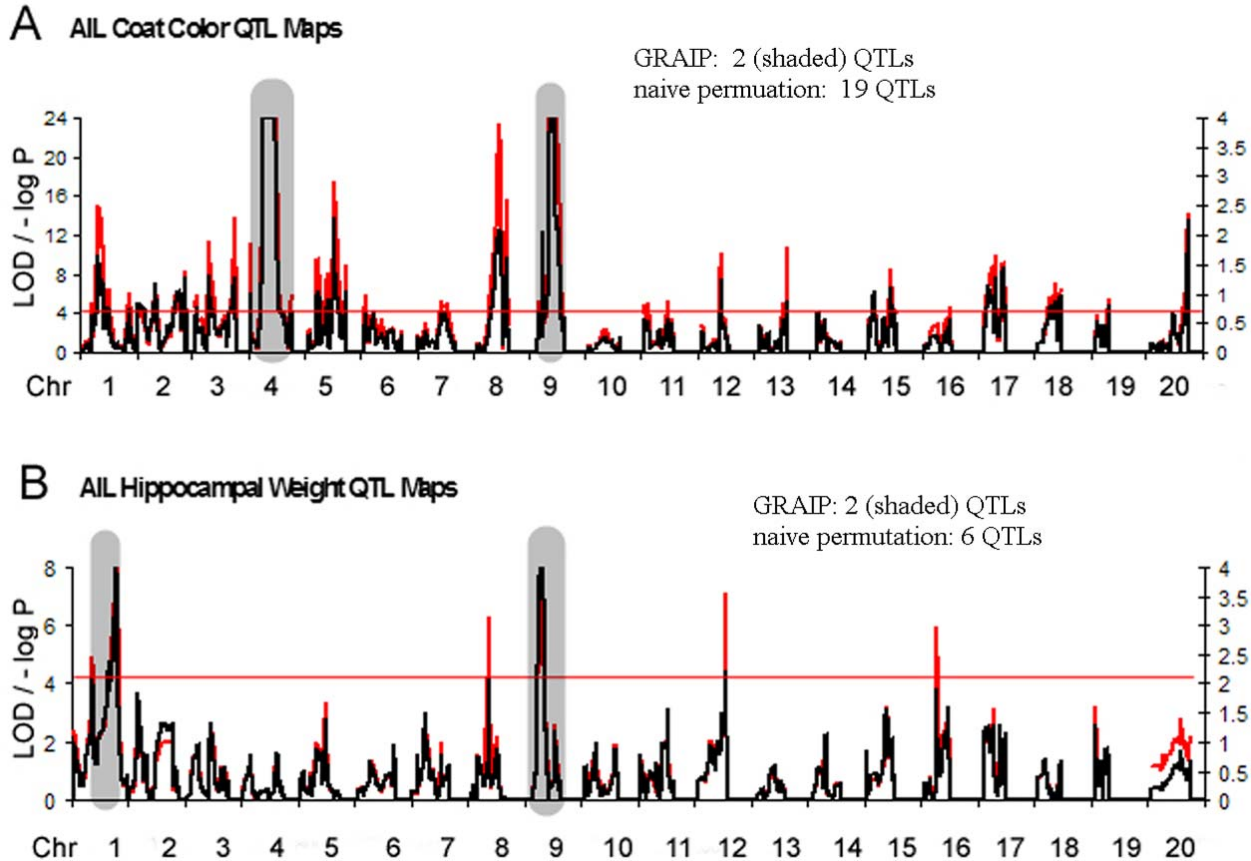
Coat color (A) and hippocampus weight (B) in the UTHSC AIL population Red traces are the simple mapping output, and the red bar is genome-wide P = 0.05 by naïve permutation. Black traces are GRAIP permutation output. Note that for ease of graphing on a -log scale we have adjusted P<1/10000 to P = 0.0001, so the maximum –log P = 4. Simple mapping results are on the left hand scale, while GRAIP results are on the right. On the Chr.4 coat color locus simple mapping value is truncated at LOD = 25, to simplify reading the graph. Shaded gray regions are significant at genome-wide P = 0.05 or better in the GRAIP results.

**Figure 4:**
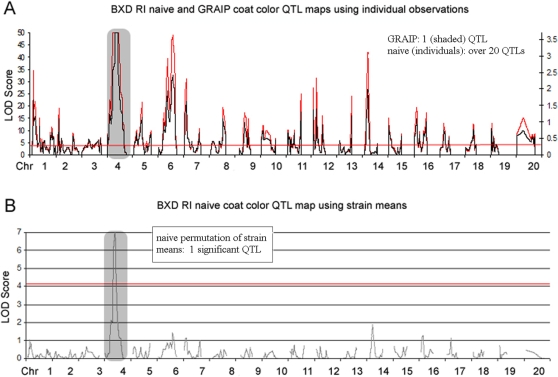
BXD coat color QTL-maps. Coat color QTL maps treating BXD observations as independent individuals versus mapping strain means. (A) Comparison of simple mapping and GRAIP for BXDs treated as individuals. Red traces are simple mapping output, and the red bar is P = 0.05 for the naïve permutation. Black traces are GRAIP mapping output (5000 permutations) and shaded gray region is significant at genome-wide 0.05 or better in the GRAIP results. (B) simple mapping output for BXD strain means. Black bar indicates P = 0.05 for the naïve (appropriate, in this case) permutation.

Using coat color and hippocampus size [12], [13] as model phenotypes with well established genetic determinants, we compared the results of a naïve QTL mapping and permutation protocol (interval maps generated and permuted for significance as if the AIL were an F2 population) with GRAIP. For coat color, the loci expected to segregate between C57BL/6J (B6) and DBA/2J (D2) has been exhaustively characterized. We expect to detect two segregating loci: the brown locus (*Tyrp1*) on Chr. 4 at 80Mb and the dilute locus (*Myo5a*) on Chr. 9 at 155 Mb. (The agouti locus does not segregate between B6 and D2.) We generated both simple (naïve permutation) and GRAIP maps in the UTHSC AIL population (Fig. 3a) as well as in the 34 C57BL/6J×DBA/2J (BXD) RI strains (Fig. 4b) available from The Jackson Laboratory (TJL) as a control.

If we examine the raw LOD scores from mapping coat color in the UTHSC AIL (Fig. 3a), using a naïve approach, it is clear that there are strong loci on Chrs 4 and 9, as expected, but there are loci with LOD scores above 4.1 (genome-wide adjusted p = 0.05 for the naïve permutation) on all chromosomes except Chr.10 (scale on left axis, trace and line indicating genome-wide significance in red). Admittedly, the strongest loci are Chrs 4 (maximum LOD score of 132) and 9 (maximum LOD score of 49) while other loci have LOD scores below 25, but since we know that only two loci are segregating, we can be relatively confident that the other loci detected as influences on coat color are false positives.

Enumeration of new QTLs in this population would therefore be impossible using a simple mapping method. Even confirmation of QTLs observed in simpler populations would be quite problematic given that, for coat color, 30% of the map is associated with a LOD score of at least 4.1, which could easily lead to mischaracterization of spurious association as confirmation of a QTL interval in a more sparsely genotyped population, at least for oligogenic traits.

In contrast, the GRAIP results for coat color mapping (Fig 3a; scale on right axis, trace in black, significant loci shaded) show significance only on Chrs 4 and 9, (genome-wide P<0.013, the minimum possible P-value with 10,000 permutations since 1.3% of permuted genome scans have at least one locus-specific P<0.0001) exactly as expected. No other loci are close to a genome-wide P<0.05.

### Mapping coat color using BXD individual observations using a naïve permutation and GRAIP

RI strains are similar to AIL strains in that within each family (i.e. strain), animals are genetically similar, albeit considerably more so than in an AIL. Treatment of RI individuals as unique yields inflated LOD scores [14] in a manner similar to AIL mapping experiments, while application of GRAIP reconstitutes mapping results in a manner similar to mapping using strain means.

We applied a slightly modified version of the GRAIP approach (constrained to produce an inbred final generation) to a set of individual observations of coat color in BXDs using a 680 animal virtual population, (34 strains, with the number of animals per strain varying randomly between 2 and 40, with a mean of 20) assigning each animal the coat color associated with the strain. Determining haplotypes of the BXD parents in the previous generation was trivial since they are the same as the BXD offspring, and recombination does not affect the outcome since the parents and offspring are inbred.

The QTL map for the 34 BXDs from TJL for coat color is quite clear—the locus on Chr. 4 is highly significant. (There are too few strains to detect the locus on Chr. 9 using our coat color encoding scheme.)

The comparison between the simple BXD coat color map based on strain means (Fig. 4b) and the simple map with naïve permutation based on individual animals (Fig. 4a; scale on left axis, trace and line indicating genome-wide significance in red) is striking. Not only is the significance of the actual QTL on Chr. 4 dramatically inflated (LOD = 140 (figure truncates at LOD = 50), up from LOD = 7 in the strain mean map) as would be expected [14] but many other QTLs, including many with extremely high LOD scores, have sprung up as well. In fact there is at least one QTL with a LOD score over 5 (genome-wide P<0.05 for the naïve permutation) on every chromosome.

Applying the GRAIP method eliminates these additional QTLs. (Fig. 4a; scale on right axis, trace in black, significant loci shaded) Only the Chr.4 QTL remains significant (P<1/5000 with 5000 permutations is a genome-wide adjusted P<0.024).

### Hippocampus weight

For examination of hippocampus weight in AILs(Fig. 3b) we focused mainly on the *Hipp1a* locus, which has been consistently identified in other populations. There are clearly other loci involved in a phenotype like hippocampus weight, and it would not be surprising for us to identify additional loci. Indeed, a glance at the simple mapping output for our AIL population indicates loci significant by naïve permutation (scale on left axis, trace and line indicating genome-wide significance in red) on Chrs 1, 8, 9, 12, and 16. The GRAIP results for the same population (scale on right axis, trace in black, significant loci shaded) only attribute genome-wide significance to loci on Chrs 1 and 9, however. Since the locus on Chr.9 has not been previously observed, except as an unpublished suggestive QTL for bi-lateral hippocampus weight (observation made using data from Lu and colleagues [13]; BXD published phenotypes record 10376 on www.genenetwork.org) we have named it *Hipp9a*. While we cannot rule out the possibility that the additional loci are also real, it is encouraging that *Hipp1a* is replicated using the GRAIP approach and interesting that there are fewer loci significant by naïve permutation with hippocampus weight (where many loci may exist) than with coat color (where only two known loci are segregating).

### How much does family matter?

**Figure 5:**
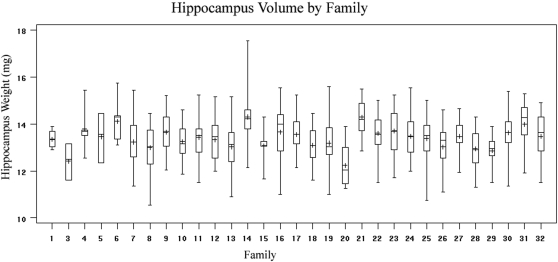
Box plot of hippocampus weight by family. Whiskers represent the distribution of the highest and lowest 25% of observations. The line across the box represents the median value, while the “+” indicates the mean. Family 2 is missing because there were not hippocampus weight observations in that group.

**Figure 6:**
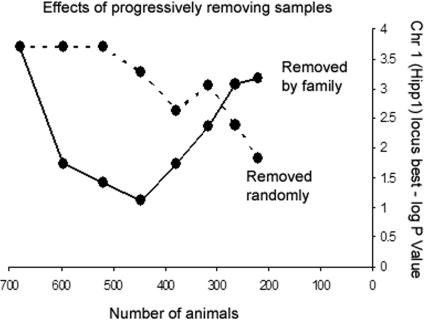
Effects of progressively removing samples from a population. Measurement were taken at the position with the best P-value in the *Hipp1a* locus. Samples removed either by family or by an equivalent number of randomly selected individuals. –log P measured at the most significant position in the original BXD data set for hippocampus weight, near the physical center of the *Hipp1a* interval.

As can be seen from the box plot of hippocampus weight, (Fig. 5) there is a significant effect of family (p<0.0001) on this phenotype, a phenomenon that we have observed for a variety of heritable characters including coat color (p<0.0001). In order to evaluate the relative importance of a large number of crosses versus sheer number of animals, we sub-sampled our observations of hippocampus weight, progressively removing either entire families or an equal number of randomly selected animals. A typical result of performing this procedure once is shown in Fig. 6, We predicted that the number of families is more important than the total number of animals to the power of the population to detect linkage. The locus specific P value, for the well-established *Hipp1a* QTL on distal Chr. 1, chosen at the point of the best P-value in the original map, decreases relatively smoothly with number of samples in the randomly diminished population. In the population where individuals are removed by family, however, the change in significance is less monotonic. This is a direct result of the family effect on the phenotype. For instance, if a given family has a high overall score in a phenotype but an allele at a given QTL that would predispose for a low phenotype, each member of the family will reduce the evidence for that QTL. In some cases (the first three sets of two families removed) the level of significance is reduced while in the last four it is increased. The removal of some families seems to have a negative effect on the significance of a given locus while removal of others has a strong positive effect, while the removal of randomly chosen animals has a much more consistently negative effect on overall significance. Choosing different families or order of families to remove consistently shows these non-monotonic effects on significance, while the effect of removing groups random individuals on significance is consistently monotonic.

### The value of locus-specific P values

**Figure 7:**
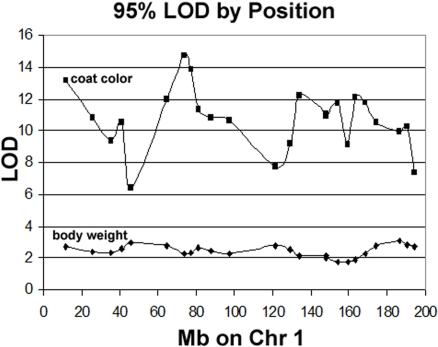
Variation in LOD score distribution by position on Chr 1. Distribution of 95^th^ percentile LOD scores by marker for 10,000 GRAIP permutations of coat color and body weight QTL mapping in the AIL population. Note that the maximum and minimum values of the 95^th^ percentile on this chromosome alone are separated by a difference in LOD of 8.3 for coat color and 1.3 for body weight, which indicates that the same LOD score is equivalent to a considerably different P value depending on position and original phenotype.

The common assumption in QTL analysis of large populations with independent observations is that any position in the genome is equally likely to be the best P-value in the genome under the null hypothesis of no linkage. Put another way, the assumption is that the P-values at all positions are identically distributed if there is no influence of genotype at that position. In the case of the UTHSC AIL population, however (Fig. 7) this is very clearly not the case. For coat color, the LOD score equivalent to a locus-specific P = 0.05 (referred to as 95% LOD) by GRAIP on Chr.1 varied from 6.4 to 14.7. For body weight the same range was 1.8 to 3.1—both considerably more varied than is typical for even medium sized F2 populations [15]. The exact origin of this variation is unclear, but we examined several possible relationships in a set of 50 permutations. In this set, variation in the LOD score equivalent to a locus-specific P = 0.05 is not correlated with the fraction of missing data at the locus. (r = −0.07, P = 0.62) for coat color as it is for small RI strain sets [15].

In order to examine whether the variation in 95% LOD scores is related to a marker's correlation with unlinked markers known to affect the phenotype (non-syntenic correlations), we calculated the absolute value of the Pearson's correlation coefficient between genotype at the major (*Tyrp1*) locus for coat color in the original data and genotype at 24 unlinked test markers on Chr.1 in data permuted using GRAIP, averaged over 1000 permutations (marker-*Tyrp1* correlation). We then calculated the Pearson's correlation coefficient between the marker-*Tyrp1* correlation and the 95% LOD score, which was significantly positive (r = 0.56, P = 0.004). In other words, GRAIP re-shuffled markers that were more highly correlated with the *Tyrp1* locus were associated with higher 95% LOD scores.

This strong positive relationship may also explain the striking difference between variation in locus-specific P-values for body weight and coat color since coat color (using our encoding scheme) has only one major determinant and one minor determinant, while body weight has many influences of small effect. As described above, the LOD score at each position in each GRAIP permutation for coat color therefore varies widely depending on its correlation with the original genotype at *Tyrp1*, the major segregating coat color locus detected using our encoding of coat color.

## Discussion

We have developed and implemented GRAIP, a permutation and simulation-based mapping approach for the analysis of AIL data, based on the idea that the identity of the parents of the final, phenotyped generation is an interchangeable unit, but the individuals in the final generation themselves, at least where the final generation expanded beyond one individual per pair of parents, are not. This concept is similar to permutation by interchanging the identity of parents used to generate a recombinant inbred intercross (RIX) population, [16], [17] though generation of haplotypes and handling of segregation in generation of the permuted population is obviously unnecessary in an RIX population, and typically phenotypic measurements are made on the RI×RI F1 means rather than being applied to individual animals.

The GRAIP approach is necessary for most AIL populations because a naïve permutation approach does not take into account the effects of family structure in the AIL population, which are important when multiple offspring from the final generation are phenotyped. A naïve permutation is appropriate for a population without substantial family structure because it permutes the relationship of individual and phenotype. When the genetic factors we are attempting to detect are confounded with family identity, however, studying linkage without respect to the relatedness of family members yields significant non-syntenic linkage. The more oligogenic the trait the more different it is between families, and the stronger apparent non-syntenic QTLs will be, though family differences are significant even in a highly polygenic trait like hippocampus weight. GRAIP addresses this problem by accounting for the family structure of the AIL cross and choosing an appropriate unit, parental identity, for permutation. It also utilizes locus-specific P-values to account for the widely varying relationship between LOD score and P-value in the AIL population, which allows us to extend the utility of the method to genome-wide QTL analysis with appropriate genome-wide adjusted significance thresholds.

Mapping of both hippocampus weight and coat color in an AIL population using GRAIP returned expected loci. In addition, analysis of hippocampus weight using GRAIP returned a novel locus on Chr.9. For coat color, where only two loci are segregating between the parental strains, novel loci would have been indicative of a serious flaw of the method to discriminate false positives from true positives, but only the expected loci were returned. This was precisely the case when a naïve permutation was compared with GRAIP for coat color. In contrast, loci significant by naïve permutation for coat color were found on nearly all chromosomes.

The approach also performed well on individual observations of RI phenotypes rather than strain means, again returning the expected coat color loci. Treating multiple RI animals as individual observations is clearly not itself a valid method of analysis [14] and is not a good choice for RI data even when adjusted using GRAIP. It is, however, a useful qualitative test of the approach.

The genome reshuffling and R/qtl mapping steps necessary to generate an original QTL map and GRAIP permutations can be executed in a day on a modern desktop computer for a small (3000-4000 permutations of a densely genotyped data set—a number sufficient to define the criteria for a genome-wide P<0.05) number of permutations and in 4–5 days for a larger (15,000–20,000) set of permutations. Naturally, for smaller sets of genotypes much more typical in AIL experiments these times will also be considerably reduced.

After applying GRAIP we used a simple mapping model to calculate the original and permuted QTL maps, which are then used to generate locus-specific P-values and genome-wide significance thresholds. Since GRAIP preserves the correlation structure, the distribution of permuted LOD statistics at each locus can be meaningfully compared to the LOD at each locus in the observed data to calculate locus-specific and genome-adjusted P-values that will ensure appropriately controlled type I error rates. The use of a more complex analysis method, such as a mixed model with random effects of parents, might improve the power to detect QTLs, and would not require modification of the GRAIP procedure itself.

### Alternate approaches and limitations of the GRAIP approach

One of the difficulties in analysis of this particular AIL pedigree was lack of complete genotype and pedigree information for all generations. Complete pedigree information, if available, would have allowed simulation of inheritance of each allele for the full pedigree. The approach we have taken accounts for relationships that come only from the final generations, and so does not fully solve the problem of uneven relatedness, for instance of members of the parental generation which was chosen as the permutable unit. We expect kinship coefficients, calculated assuming the parents are unrelated, will not be too much smaller than the true kinship coefficients, and so our approach accounts for the majority, but not all, of the relationship problem.

Assuming a lack of complete pedigree information, a intuitive alternative approach would be to permute phenotype within sibship. Unfortunately, this approach does not very fully break the association between genotype and phenotype. Siblings have more similar genotypes and also more similar phenotypes, and so real evidence for linkage would still appear in data permuted in this way. Likewise, permutation of genotype within sibship or regeneration of genotypes within sibship from parental haplotypes without permuting parental identity does not fully break the genotype/phenotype association.

### The usefulness of AILs for mapping of QTLs

The most broadly applicable question related to AILs is whether they are a good population for fine mapping QTLs. Of particular concern are differences in the extent of non-syntenic correlation with observed markers, since this could affect the relative significance of neighboring and distant markers and bias the interpretation of mapping position. While AILs generated with larger numbers of smaller families, rotated breeding in the final generation, and a larger number of founders will very likely suffer much less severely from the reduction in power due to family structure, in our view the fundamental problem of fixation of loci within families and non-independence of samples remains as long as more than one animal per cross in the final generation is phenotyped, and these issues could substantially interfere with estimation of the QTL confidence interval. Although researchers have narrowed identified regions in AIL crosses that have subsequently shown overlap with congenic analyses [4], it is unclear to what extent this reflects widespread utility of the method.

While we have found our AILs quite valuable as a founder population for a set of new, highly recombinant BXD RI strains there are better methods for generating useful and highly recombinant RI strains with two [18] or more [19], [20] progenitor strains that result in full independence of recombination events between strains, so this use in itself probably does not constitute a reason to generate a high-generation AIL.

Alternative strategies such as large F2 crosses, RI mapping experiments with larger mapping panels [18], [21] especially in conjunction with transcriptome-QTL and genetic correlation approaches [22], [23], RIX crosses, [24] or construction of congenics [25], offer more rapid turnaround and, except perhaps in the case of the RIX, much simpler analysis procedures.

The RIX population deserves particular mention here. Because it is based on intercrossing RI lines, which have several times the recombination density of F2 crosses, (especially if constructed using RI lines generated by inbreeding of AIL progeny) it offers a rapid means of generating a non-inbred population with a high density of recombination—essentially the goal of creating an AIL population. Since RI lines are in general already densely genotyped, balanced crosses emphasizing recombinations in areas of interest are relatively straightforward to construct and can be analyzed in combination with parental RI lines. In addition, RIX crosses have a distinct advantage over AILs in that they share with RIs the property of being a reference population [23]. Thus multiple animals can be phenotyped to reduce variation or examine the genetic relatedness of multiple phenotypes.

### Recommendations on making an AIL population in mice and other populations

The GRAIP approach is designed to account for the effects of family structure on the phenotypes of an AIL population. Minimizing these effects reduces the differences we would expect between GRAIP and a naïve permutation approach, which immediately suggests several recommendations for improving AIL populations.

We believe that the issues described here in mice should apply to AILs developed in other organisms, where the final generation is expanded, but in organisms whose breeding characteristics allow large populations in intermediate generations and do not require expansion of the final generation to create enough individuals for QTL mapping projects, for instance Arabidopsis or maize, the issues we discuss can be alleviated.

Since this approach is not always feasible, subject to the relative costs of breeding and phenotyping, we would recommend expanding the AIL population 1-2 generations before the final generation of an AIL and making sure to rotate parents frequently in the process of generating the final generation. An AIL population based on 150 distinct families with 6 offspring each is likely to be a more powerful mapping population than one based on 30 crosses with 30 offspring each though in both cases 900 animals are being phenotyped.

The approach taken by Iraqi and colleagues [6] of dramatically expanding the set of parents of the final generation and using a small number of animals per family should also dramatically improve the power of AILs as a mapping population. The small effect of randomly removing sample size shown in Figure 6 suggests that analysis of large numbers of animals from each family is relatively inefficient, though as in the case of RI populations this may be less the case when heritability is low.

Additionally, we would suggest retaining DNA samples from each member of each generation of the cross and, again subject to the cost of phenotyping, considering generating phenotypes for parents and grandparents of the final generation. It is possible that more complex models may allow retention of more information as in similar populations with more extensively retained histories [26] , and at a minimum retention of DNA samples at each generation will dramatically simplify determination of haplotypes in the final generation.

Finally, we would also recommend testing only loci that are well established using other, simpler populations and analyzing data using the GRAIP method rather than applying a simple mapping method. These precautions will improve the likelihood that fine mapping results using an AIL population represent genuine improvements in the location of genes underlying quantitative loci.

### Summary

AILs are an interesting approach to the problem of fine mapping, but generation of an AI population with strong mapping potential is more dependent on careful design of the breeding and testing populations than on sheer number of animals produced. Family structure is a serious problem with implications for published and future studies using this type of population and must be taken into account both at the design and analysis stages in order to avoid frequent false positive results and bias in identification of exact QTL positions, which will simply look like unexpectedly good fine mapping results in the absence of more genome-wide genotype data. The AIL approach is a potentially valuable method for fine mapping, provided care is taken in the generation of the mapping population. It is particularly important that family effects in the final generation be minimized by using a few individuals from any particular cross in the final generation—one offspring per cross is ideal!—and to generate the final phenotyped generation using as many crosses as possible.

## Methods

### Intercross and RI strain breeding and care

C57BL/6J (B6) and DBA/2J (D2) male and female animals were purchased from TJL (Bar Harbor, ME) and bred at UTHSC in a specific pathogen free (SPF) facility to generate B6D2F1 and D2B6F1 animals, which were intercrossed to generate an F2 population as described previously [27]. All animals, parental, BXD, and all of the AIL families considered in this paper were bred and cared for according to the animal care and husbandry guidelines of UTHSC.

Commercially available BXD strains were purchased from TJL and bred in-house as required. These strains were generated by repeatedly intercrossing offspring of B6 and D2 parental strains in the mid-1970s (BXD1 through BXD32) and 1990s (BXD33 through BXD42) by Benjamin Taylor and colleagues [28], [29].

### Advanced intercross generation

B6 and D2 male and female animals were purchased from TJL and bred at UTHSC in a specific pathogen free (SPF) facility to generate B6D2F1 and D2B6F1 animals, which were intercrossed to generate an F2 population. For all breeding following the F2 generation, two males and two females were placed in a breeding cage. When a female was observed to be pregnant, the other animals were removed from the cage. Approximately 30 breeding cages were maintained at each generation beyond F2. Animals from the F2 pool were randomly chosen and mated to create a F3 population in this manner. Following F3, breeding followed a version of the advanced intercross technique described by Darvasi and Soller [3]. Instead of random breeding, however, matings were chosen at each generation to minimize the number of common parents by examining common ancestors in the third generation prior to the generation being set up. Breeding partners were chosen manually so that there was no more than one common ancestor in the previous three generations.

### Genotyping

Genotyping of our F2 [27] and the BXD RI strains [30] strains has been described elsewhere. For the advanced intercross a total of 329 microsatellite loci polymorphic between B6 and D2 strains and distributed across all autosomes and the X chromosome were amplified and typed. 329 loci gives a resolution of approximately 4.6 cM on the unexpanded map of the mouse genome. Since Darvasi and Soller [3] estimated a n/2 expansion and we were genotyping 10^th^ generation AIL individuals we expected a post-expansion genotyping density of 22.8 cM. Given the largen and uneven family sizes in the final generation, however, the observed expansion was extremely uneven, though average expansion was similar to expectation. Markers only a few cM apart were apparently unlinked, while other regions showed no expansion or even contraction. This observation is likely to be exclusively an artifact of the final generation expansion.

Genotypes were provided for all 1333 10^th^ generation AIL mice courtesy of the Mammalian Genotyping Service (MGS; National Heart, Lung, and Blood Institute (NHLBI) Contract Number HV48141), and the authors would like to express their gratitude to Dr. James Weber and colleagues for this extensive and valuable data set. Genotype files are currently available online at www.nervenet.org/mmfiles/mmlist.html. Physical positions were taken from the May 2004 University of California, Santa Cruise (UCSC) mouse genome assembly (mm5), which used data obtained from the Build 33 assembly by the National Center for Biotechnology Information (NCBI).

### Phenotyping

We used hippocampus weight and coat color as example phenotypes. Coat color was assessed in the entire advanced intercross while hippocampus weight was only measured in 679 animals. Measurement of these weights has been described previously [13]. We used a numerical scale to encode coat color (0 = black, 1 = gray, 2 = brown, 3 = DBA (dilute, brown, non-agouti)) as a “darkness of coat” observation. This is a convenient method of capturing the dilute (*d*) and brown (*b*) loci, which segregate between B6 and D2. The coat color loci segregating between B6 and D2 have known molecular determinants: tyrosinase-related protein 1 (*Tyrp1*; Chr.4: 79.1 Mb; also known as the brown (*b*) locus) and myosin 5a (*Myo5a*; Chr.9: 75.4 Mb; also known as the dilute (*d*) locus).

Because of the encoding, we expect the brown locus (phenotypes 0 and 1 have the black allele, while phenotypes 2 and 3 have the brown allele) to be more strongly detected than the dilute locus (phenotypes 0 and 2 have the non-dilute allele, while phenotypes 1 and 3 have the dilute allele), depending on the exact number of animals with each coat color. This combination of what are actually multiple separate effects into a single scale is typical of complex traits, where the multiple contributions of separate genes are almost always collapsed along a single axis.

### Estimation of parental haplotypes

Parental haplotypes were estimated using SimWalk 2.6 [31], [32], selecting at most 13 siblings per family for speed of computation and considering one chromosome at a time. Where the software was unable to predict to which strand a particular allele belonged, we assigned allele to strand so as to minimize number of recombination events. In regions where both parents were heterozygous the phase was often ambiguous. Provided that individual genotypes in the penultimate generation can be accurately inferred from the final generation, however, these haplotypes are sufficient for simple single marker association because analysis occurs only at the marker and does not require flanking marker status. Inclusion of grandparental identity and thereby relations among families helps to reduce uncertainty, but simple single marker-only mapping is still preferable if the density of markers is sufficient. All haplotypes used are available at http://www.nervenet.org/papers/GRAIP.html.

### Genome reshuffling for AI permutation (GRAIP) genotypes

Since the unit to be exchanged is the identity of the parents of the final, phenotyped generation, we first shuffled the identities of individuals within sex in this generation. Next we generated virtual gametes and combined them to create the genotypes of the permuted final AIL generation. Briefly, the number of chiasmata per chromosome in each gamete is determined by drawing from a Poisson distribution with mean equal to the length of the chromosome in Morgans. Positions of the chiasmata are drawn from a uniform distribution of the length of the chromosome, iteratively thinned to ensure a maximum of one recombination in a given distance (default is 10 cM) centered around a given chiasma. This estimate is slightly low because simulating interference in this manner lowers the mean number of recombinations. However, even separate runs with two-fold variation in recombination rates were extremely similar. This is expected since we are estimating association only at actual marker positions. We are using standard MGI (Mouse Genome Informatics) estimates of genetic position, not AIL-based calculated genetic positions which are highly biased by repetition of early recombinations in the population, to estimate missing physical positions (below). Recombination positions could also be simulated using a more sophisticated model [33].

A starting haplotype is then randomly chosen and the script imposes the recombination pattern on the marker positions and switches between haplotypes as needed to simulate recombination. Since sex is assigned to each generated individual, the X chromosome from the father is either assigned as an intact, randomly chosen haplotype (for females) or treated as null (for males), and the genotype of the X chromosome in the zygote is generated appropriately. The output of this process is a set of genotype files each containing the set of genotypes of a permuted AIL population.

### Mapping

The original population, naïve permutations, and GRAIP permuted genotypes were treated identically with respect to generation of QTL maps. Since the AIL we are using as a test population is relatively densely genotyped we mapped using regression at marker locations in all cases, which also had the effect of halving the time required and eliminating potential worries about the accuracy of assigning genotype probabilities between markers. We used the multiple imputation method of Sen and Churchill [34] as implemented in the R/qtl package for the R environment [35] to handle missing data, imputing 16 sets of genotypes per map. (No significant differences were observed between output using 16 and 64 imputations, and mapping results omitting imputation are also quite similar.) The X chromosome was handled as if it were an autosome since the mapping model is designed for an F2 population and requires identity of the cross direction to handle X chromosome data correctly, so loci on this chromosome should be treated with care. Genome-wide adjusted significance thresholds for naïve permutations were generated by the method of Churchill and Doerge. [36]

### Generating locus-specific P-values for physical positions

We permuted AIL parental identities, regenerated permuted final AIL generations, and performed QTL mapping for all permutations using the mapping protocol described above. Once all GRAIP permuted maps were generated, we calculated locus-specific p-values for each marker for the original data using the observations from the permuted genome maps at each locus as a null distribution. While locus-specific P-values derived from permutations are not crucial for all populations, the AIL family structure causes dramatic variation between LOD score at a given locus and corresponding P-value.

We describe determination of locus specific P-values and discuss their usefulness for different data sets in detail elsewhere [15]. Briefly, at every locus j we compare L(j), the LOD score at that locus in the original data with L*(i,j), the LOD scores at locus j in each of i permuted data sets. The locus-specific P-value, P(j) is the proportion of L*(i,j) ≤L(j). The distribution of locus specific P-values was uniform for randomly generated phenotypes in which a family bias was chosen and individual phenotypes selected from a normal distribution around the family-biased mean.

P-values at regular intervals were interpolated based on the known physical and genetic positions of markers. We linearly interpolated missing physical positions using flanking markers with known physical and genetic positions and linearly interpolated P values on a regularly spaced set of physical positions.

### Generating genome-wide adjusted P-values

We computed locus specific P-values, P*(i,j), for each L*(i,j) in the manner described above where L(j) is L*(j) for a fixed i^th^ GRAIP permutation. Genome-wide adjusted P-value at locus j, Pa(j), can then be computed by creating an ordered list, MP*, of the minimum P-value from each GRAIP permuted map. Then Pa(j) is the proportion of MP*≤P(j). This is similar to Churchill and Doerge [36] with the addition of explicit conversion of best permuted observation (typically expressed as a LOD score) in each genome scan to a locus-specific P-value.

Our implementation varied slightly from the procedure described above in the final step. To generate MP* we sampled with replacement at least 1000 times from our available set P*(i) genome scans in order to maintain consistency with our general approach to combining multiple data sets, described elsewhere [15]. For large samples these methods produce essentially the same MP* distribution.

### Applying GRAIP to BXD RI data

As previously introduced, the BXD strains are similar to AIL strains in that a population of BXD strains is usually organized into a set of individual offspring. Since these offspring are inbred, a phenotypic mean is often associated with a single copy of each strain's genotype to reduce non-genetic noise. We created two virtual populations of BXD RI animals using the 34 strains from The Jackson Laboratory and mapped coat color using three approaches, each employing R/qtl as described. We first associated our coat color phenotype with a single genome for each strain as described above.

Next we generated a virtual population of 680 BXD RI animals, with the number of animals per strain as a random, uniformly distributed even number between 2 and 40, with a mean of 20. We generated GRAIP-shuffled genomes for these populations as described above with the slight modification that male and female parent identities were permuted together, since in this case the parents are themselves are constrained to be inbred.

### Testing the effects of family structure on QTL significance

In order to provide an example of the effects of eliminating different segments of the population on a more complex phenotype, we examined hippocampal weight, a phenotype we have measured and published on before [12], [13] which has a particularly reliable QTL on distal Chr.1, which is also detected in the AIL population. In order to demonstrate the effect of families on QTL significance, we progressively removed either entire crosses or an equivalent number of randomly chosen individuals from the original population (31 crosses, 679 individuals). We evaluated significance using the locus-specific P value, evaluated at a marker estimated to be near the middle of the well-established *Hipp1* interval on distal Chr. 1 on a physical scale.

### Software

All software described is available at http://www.nervenet.org/papers/GRAIP.html. For the analyses above, we wrote a script, **GRAIPGeno.py**, using Python 2.4 to generate permutations of final generation genotypes. Since each GRAIP-based permutation requires generation of a complete QTL map, we wrote a simple R [37] script, **GRAIP.R**, to automate the process, which was performed using R/qtl [35]. Parsing of mapping output files from R/qtl, generation of locus-specific P-values, and generation of genome-wide adjusted P-values were all handled by Python scripts described elsewhere [15].
